# Medical Classes

**Published:** 1848-05

**Authors:** 


### MEDICAL CLASSES.

The University of Pennsylvania stands first, this year, among American schools of Medicine in respect to the size of its class, which numbered, according to the catalogue, 508. Of these 47 were graduates of that and 18 other institutions. The Jefferson Medical College ranks next to the University, the catalogue showing that the number of its students was 480, of whom 58 were graduates. Not having seen a catalogue of the University of New York we cannot say whether that or the University of Louisville comes next. Of the 406 students in attendance at the latter, 13 were graduates. The catalogue of the medical department of

Transylvania University embraces the names of 167 students, of whom 5 were graduates, and 17 were students in the Academical department and attended only the chemical lectures. The class of the Starling Medical College numbered 138, of whom 4 were graduates. The number of medical students in the Western Reserve College was 240, three of whom were graduates. The catalogues of the other medical schools of the country have not yet been received.

